# Energy Levels of Singly-Ionized Platinum

**DOI:** 10.6028/jres.097.004

**Published:** 1992

**Authors:** Jean Blaise, Jean-François Wyart

**Affiliations:** Laboratoire Aimé Cotton,^1^ Bât. 505, C.N.R.S. Π, Centre Universitaire, F-91405-ORSAY ( France)

**Keywords:** atomic spectroscopy, electronic configurations, energy levels, platinum

## Abstract

The analysis of Pt II is extended by using accurate wavelength measurements by Sansonetti et al. Forty-three new even and 104 new odd levels have been found. The Slater-Condon parametric method is used for the interpretation of the 5*d*^9^, 5*d*^8^6*s*, and 5*d*^7^6*s*^2^ low even configurations and the 5*d*^8^(7*s*+6*d*) high even configurations with root mean square deviations smaller than 80 cm^−1^. The importance of the 5*d*^8^–5*d*^7^6*s* core interaction in interpreting the even-parity levels is stressed.

## 1. Introduction

The spectrum of platinum emitted by a hollow cathode lamp has been recently observed and measured [1]. The improved wavelengths of the classified lines led Reader et al. [2] to determine accurate energies for the known levels. The extensive line list comprised many unclassified lines. Their interpretation has been undertaken at Laboratoire Aimé Cotton in order to improve the knowledge of excited levels at the end of the 5*d*-period.

The strong unclassified lines have been interpreted in the present work with the support of theoretical energy level predictions and a computer program to search for recurring energy differences in the list of observed wave numbers. The measured wave numbers of classified lines deviate from the differences between their initial and final levels by less than 0.050 cm^−1^ if the lines are not blended with other transitions. The energy levels are reported in Tables 2, 3, and 5, in which the 3-digit values are taken from Ref. [2]. The *J*-values of some levels have been changed and the newly classified lines led to slight modifications of their energies. The uncertainty of the levels depends on the intensities and spectral regions of their transitions. It ranges from 0.050 to 0.100 cm^−1^. The classified lines are reported in Ref. [1].

## 2. Interpretation of the Low Even-Parity Configurations 5*d*^9^, 5*d*^8^6*s*, and 5*d*^7^6*s*^2^

In 1977 [3], a systematic description of the even configurations (5*d* + 6*s*)*^N^* was performed in the framework of the Slater-Condon parametric method. It was shown that configuration mixing was very important within these groups and led to the revision and limited extension of some analyses. In the absence of definite configuration assignments for many levels, the sum of the squared amplitudes represented 53% of all 5*d^N^* levels from Lu II to Au II, 56% for 5*d^N^*^−1^6*s* and only 27% for 5*d^N^*^−2^6*s^2^*. In Pt II, all 21 levels found by Shenstone [4] were supported by the theoretical calculation, but six of his empirical *LS* designations did not correspond to the leading component of the eigenfunction. The 5*d*^7^6*s*^2^-configuration was limited to four known levels and the relevant energy parameters needed to be fixed or constrained. The present analysis was guided by the results of [3] and the number of levels of the (5*d* + 6*s*)^9^ group has been brought from 21 to 33. The present interpretation of the three configurations 5*d*^9^, 5*d*^8^6*s* and 5*d*^7^6*s*^2^ leads to improved parameter values, a number of constraints in the least-squares fitting process being now removed. The present set of parameters includes: a constant energy for all three configurations, *A*, the energy differences between configurations, *S*(*d*^9^−*d*^7^*s*^2^) and *S*(*d*^8^*s*−*d*^7^*s*^2^), all Slater integrals describing the electrostatic interactions within the studied group, the effective electrostatic parameters *α*_0_ and *β*_0_ as defined in the formalism of orthogonal operators [5], and finally, the usual spin-orbit parameters. These 18 parameters have been reduced to 13 adjustable ones by means of constraints detailed in Table 1. These constraints were derived from earlier studies of (5*d* + 6*s*)*^N^* groups. The root mean square deviation is 73 cm^−1^. The comparison of experimental and theoretical energies is given in Table 2. The theoretical data are limited to the theoretical energy *E*_th_, the first component of the eigenfunction and the percentage of the components of the 3 configurations (squared amplitudes) in the eigenfunctions. The coefficients of the interaction parameter *R*^2^(5*d*^2^,5*d*6*s*) in intermediate coupling show that 5*d*^8^6*s*^2^*P*_1/2_ and 5*d*^7^6*s*^22^*P*_1/2_ which are distant by 33700 cm^−1^ have a mutual repulsion of 7000 cm^−1^ and that four other levels of 5*d*^8^6*s* are shifted to lower energies by more than 2000 cm^−1^.

## 3. The Predicted Low Configurations of Pt III

The spectrum of Pt III is still unknown but, for application to Pt II, its low energy levels can be predicted by means of the Slater-Condon method. By comparing the lowest energy levels of *5d^N^*, 5*d^N^*^−1^6*s* and 5*d^N^*^−2^6*s*^2^ in Hf III (*N* = 2) [6], W III (*N* = 4) [7], Au III (*N* = 9) [8] and Hg III (*N* = 10) [9], one can reasonably assume that the excitation energies of *5d*^7^6*s* ^5^F_5_ and *5d*^6^6*s*^2 5^*F*_5_ and 5*d*^6^6*s*^2 5^D_4_ levels above the ground level 5*d*^8 3^F_4_ are about 20000 and 60000 cm^−1^, respectively. All other parameters needed for describing (5*d*+6*s*)^8^ in Pt III may be obtained from regular trends investigated in second spectra [3] and in third spectra. The results of this preliminary study are summarized below.

For all *J*-values, the configuration 5*d*^8^ does not overlap the energy range of the 5*d*^7^6*s* and 5*d*^6^6*s*^2^ configurations, but this does not prevent configuration mixing. The effect of 5*d*^6^6*s*^2^ is a constant shift of about −800 cm^−1^ for all levels of 5*d*^8^ except ^3^P_0_ and ^1^S_0_, both shifted by −1400 cm^−1^. The effect of the 5*d*^7^6*s*−5*d*^8^ mixing is more selective and is reported in the last column of Table 3. These shifts mean that the 5*d*^8^ parameters would certainly differ if fitted in the approximation of *isolated* configurations or in mixed groups (5*d*+6*s*)*^N^*. The *LS* names are well defined except for the *J* = 2 levels, for which ^3^P_2_ is nowhere the leading component of the eigenfunction. Since the second and third *J* = 2 levels have respectively dominant ^3^F_2_ and ^1^D_2_ characters, the lowest *J* = 2 level has been given the designation ^3^P_2_ for identification purposes in the next step of the work.

## 4. Interpretation of the Upper Even Configurations

Nine high even levels were identified by Shenstone [4] as 5*d*^8^7*s* and 5*d*^8^6*d*. One of these levels has now been rejected and the *J*-values of two revised. The three levels of 5*d*^8^8*s* and 5*d*^8^7d have not been confirmed. Thirty-two levels have been found between 101500 and 121700 cm^−1^. The intensity of their transitions and some relatively large deviations *E*_exp_−*E*_th_ in the separate studies of these configurations led us to evaluate their mixing. The 21 integrals needed to describe the levels of 5*d*^8^7*s*+5*d*^8^6*d* were reduced to 15 adjustable parameters by means of constraints given in Table 1. The mixing of the lowest *J* = 1/2 levels leads to a well-defined value for the interaction parameter *R*^2^(5*d*7*s*, 5*d*6*d*) and the final rms deviation is 79 cm^−1^. As shown in Table 1, the values of the parameters *F*^2^(5*d*,5*d*) and *α* for 5*d*^8^(6*d* + 7*s*) differ significantly from those for 5*d*^7^6*s*^2^ and 5*d*^8^6*s*; however, the parameters are well-defined in the least-squares fit. We consider this to be an effect of truncation problems discussed in Sec. 3. It seems likely that these inconsistencies would be corrected if all six configurations (5*d* + 6*s*)^8^7*s* + (5*d* + 6*s*)^8^6*d* were studied together. This extended parametric study has not been undertaken because 5*d*^6^6*s*^2^7*s*, 5*d*^6^6*s*^2^6*d* and 5*d*^7^6*s*6*d* are totally unknown and only two levels of 5*d*^7^6*s*7*s* are located so far. The predictions of our restricted study might well be unreliable and the theoretical energies of unknown levels have therefore not been reported here.

## 5. Odd Levels of Pt II

The lowest odd levels were attributed to 5*d*^8^6*p* by Shenstone [4]. This configuration is also known in other ions of the isoelectronic sequence through Bi VII [9–11]. The approximation of an *isolated* 5*d*^8^6*p* configuration, if valid, has been used for the theoretical study of Au III-Bi VII spectra. It does not hold for Pt II. In second spectra, the overlap of 5*d^N^*6*p*, 5*d^N^*^−1^6*s*6*p* and 5*d^N^*^−2^6*s*^2^6*p* requires a multiconfigurational treatment. In Hf II, Ta II, W II, Au II and Hg II [12], these low odd configurations had been interpreted with rms deviations smaller than 200 cm^−1^. For unclear reasons, the rms deviation for Pt II is larger than 500 cm^−1^ and the designations reported in Table 4 are carefully limited to the lowest levels. Some of them might well be revised with further advances in the parametric interpretation. The 5*d*^7^6*s*6*p* configuration starts with the 62820 level, for which we explain the absence of decay to 5*d*^7^6*s*^2 4^F_9/2_ by the selection rule on the strongly forbidden transition 6*s*6*p* ^3^*F*_0_−6*s*^2 1^*S*_0_. Most of the levels without designation belong to 5*d*^7^6*s*6*p* with some admixture of 5*d*^6^6*s*^2^6*p* for the highest energies.

## 6. Conclusion

The strongest unclassified lines of Pt II have been interpreted by extending the early analysis of Shenstone with the help of accurate wavelength measurements and parametric calculations of the main configurations. The number of levels has been brought from 29 to 72 in the even parity and from 71 to 174 in the odd parity. The theoretical study stresses the importance of the 5*d*^8^ − 5*d*^7^6*s* interaction and, although somewhat preliminary, the parametric interpretation of the low odd levels indicates that all levels with *J* = 3/2 through 11/2 below 79000 cm^−1^ have been found.

## Figures and Tables

**Table 1:** Fitted energy parameters (cm^−1^) of the even configurations of Pt II. Standard deviations of the parameters are given in parentheses

Parameter	5*d*^7^6*s*^2^	5*d*^8^6*s*	5*d*^9^	5*d*^8^6*d*	5*d*^8^7*s*
*A*	58028 (65)			121854 (119)	112271 (66)
*S*(5*d*^8^6*s*−5*d*^7^6*s*^2^)		−30621 (94)			
*S*(5*d*^9^−5*d*^7^6*s*^2^)			−51804 (117)		
*F*^2^(5*d*,5*d*)	52391 (219)	50566 (202)		46155 (235)	46155 g
*F*^4^(5*d*,5*d*)	39365 (318)	38754 (35)		39579 (541)	39579 g
*F*^2^(5*d*,6*d*)				3544 (369)	
*F*^4^(5*d*,6*d*)				1252 (405)	
*G*^0^(5*d*,6*d*)				767 (46)	
*G*^2^(5*d*,6*d*)				1256 (227)	
*G*^4^(5*d*,6*d*)				1256 e	
*G*^2^(5*d*,6*s*)		15354 (180)			
*G*^2^(5*d*,7*s*)					1879 (247)
*R*^2^(5*d*^7^,6*s*^2^)		16889 b			
*R*^2^(5*d^2^*,5*d*6*s*)	−20905 (242)h	−20277 c			
*R*^2^(5*d*6*d*,5*d*7*s)*				2568	(302)
*R*^2^(5*d*6*d*,7*s*5*d*)				942	(657)
*α*_0_	15.1 a	15.1 (4.5)		115 (6.5)	115 g
*β*_0_	−204 a	−204 (50)		−204 f	−204 f
ζ_5_*_d_*	4607.1 (19)	4349.5 (21)	4092.0 d	4378 (18)	4335 (27)
ζ_6_*_d_*				228 (17)	

a Parameters constrained to be equal in 5*d*^7^6*s*^2^ and 5*d*^8^6*s*.

b The parameter *R*^2^(5*d*^2^,6*s*^2^) of the 5*d*^7^6*s*^2^−5*d*^9^ interaction is held in a constant ratio with the *G*^2^(5*d*,6*s*) of 5*d*^8^6*s*.

c Slater parameters R^(2)^(5*d*^2^,5*d*6*s*) for 5*d*^8^6*s*−5*d*^9^ and 5*d*^8^6*s*−5*d*^7^6*s*^2^ interactions are held in a constant ratio.

d ζ(5*d*^7^6*s*^2^) + ζ(5*d*^9^)=2ζ(5*d*^8^6*s*).

e *G*^2^(5*d*,6*d*) = *G*^4^(5*d*,6*d*).

f Held fixed to the fitted value of the lowest configurations.

g Held equal to the same parameter in 5*d*^8^6*d*.

h Parameter for the 5*d*^7^6*s*^2^−5*d*^8^6*s* interaction.

**Table 2:** Low even energy levels of Pt II. The theoretical energies *E*_th_ are those of the mixed configurations 5*d*^9^,5*d*^8^6*s* and *5d*^7^6*s*^2^ (designated A, B, and C in the first components of the eigenfunction)

E_exp_ (cm^−1^)		*J*	*E*_th_ (cm^−1^)	First comp.%		5*d*^9^ %	5*d*^8^6*s* %	5*d*^7^6*s*^2^ %
	0	5/2	16	A ^2^D	90.7	90.7	7.6	1.7
	4786.611	9/2	4862	B ^4^F	96.6	0	100.	0
	8419.822	3/2	8475	A ^2^D	62.9	62.9	34.5	2.6
	9356.274	7/2	9234	B ^4^F	67.5	0	99.6	0.4
	13329.227	5/2	13345	B ^4^P	36.2	5.5	94.1	0.4
	15791.276	3/2	15639	A ^2^D	32.3	32.3	65.1	2.6
	16820.894	5/2	16770	B ^4^F	60.0	0.4	99.3	0.3
	18097.715	7/2	18171	B ^2^F	63.8	0	98.8	1.2
	21168.684	3/2	21146	B ^4^F	39.5	0.1	94.1	5.8
	21717.260	1/2	21774	B ^4^P	87.4	0	99.8	0.2
	23461.503	5/2	23542	B ^2^F	48.3	1.2	95.8	3.0
	23875.553	3/2	23886	B ^4^P	56.3	0.3	87.4	12.3
	24879.480	9/2	24846	C ^4^F	67.7	0	13.6	86.4
	27255.687	1/2	27207	B ^2^P	77.8	0	84.7	15.3
	29030.479	7/2	28968	B ^2^G	88.0	0	94.5	5.5
	29261.967	9/2	29341	B ^2^G	78.6	0	81.3	18.7
	32237.007	3/2	32182	B ^2^D	53.3	3.4	85.6	11.0
	32918.561	5/2	32981	B ^2^D	36.2	1.4	87.3	11.3
	34647.221	7/2	34624	C ^4^F	95.2	0	1.5	98.5
	36484.028	5/2	36555	C ^4^F	55.6	0.1	9.4	90.5
	37877.792	3/2	37895	C ^4^F	43.7	0.1	12.9	87.0
N	41434.11	5/2	41433	C ^4^P	76.1	0.1	0	99.9
N	42031.85	3/2	41986	C ^4^P	50.1	0	11.9	88.1
N	43737.40	9/2	43774	C ^2^G	52.7	0	4.5	95.5
N	46046.43	1/2	46086	C ^4^P	76.6	0	9.0	91.0
N	48591.04	11/2	48524	C ^2^H	100.	0	0	100.
N	50564.60	7/2	50607	C ^2^G	79.8	0	4.2	95.8
		1/2	53204	B ^2^S	76.6	0	86.6	13.4
N	53749.63	3/2	53722	C ^4^P	50.1	0	3.8	96.2
N	54373.47	5/2	54333	$C{\;}_{3}^{2}D$	53.5	0.1	2.5	97.4
N	58062.04	5/2	58072	C ^2^F	81.7	0.1	3.9	96.1
N	58491.21	9/2	58518	C ^2^H	68.1	0	0.6	99.4
N	60986.75	1/2	60939	C ^2^P	65.8	0	20.0	80.0
N	64003.90	7/2	64001	C ^2^F	83.7	0	1.3	98.7
		3/2	65221	$C{\;}_{3}^{2}D$	50.8	0.2	4.3	95.5
		3/2	77750	$C{\;}_{1}^{2}D$	88.5	0.9	0.4	98.7
		5/2	79860	$C{\;}_{1}^{2}D$	67.0	0.5	0.1	99.4

Note: N—new energy level.

**Table 3:** Energy levels of Pt III 5*d*^8^ predicted in the parametric study of (5*d* + 6s)^8^

*J*	Energy cm^−1^	*5d*^8^ purity %	First comp. %		Second comp. %		Third comp. %		Shift (cm^−1^) *d*^7^*s*−*d*^8^
4	0	98.8	^3^F	94.7	^1^G	4.0			−800
2	5547	94.4	^1^D	41.1	^3^P	37.7	^3^F	16.3	−3350
3	9859	98.4	^3^F	98.4	(^2^F)^3^F	1.1			−1000
2	14249	93.2	^3^F	47.6	^3^P	40.6	^1^D	5.1	−3050
0	15127	93.4	^3^P	81.5	^1^S	11.9	(^2^P)^3^P	5.5	−3800
1	16700	89.7	^3^P	89.7	(^2^*P*)^3^P	8.4			−4950
4	21675	89.8	^1^G	86.0	(^2^G)^1^G	5.8	^3^F	3.9	−2850
2	24760	92.5	^1^D	48.0	^3^F	33.9	^3^P	10.7	−3200
0	46301	60.3	^1^S	58.3	(^2^P)^3^P	28.0	(^4^P)^3^P	10.6	−1350

**Table 4:** Upper even levels of Pt II. The theoretical energies *E*_th_ are from the parametric study of 5*d*^8^6*d*+5*d*^8^7*s*. The core term of 5*d*^8^ is indicated in parenthesis for 5*d*^8^6*d* only

	E_exp_ (cm^−1^)	*J*	*E*_th_ (cm^−1^)	Designation	5*d*^8^7*s* %	5*d*^8^6*d* %	Leading *LS* comp. %	
	95803.363	9/2	95837	(^3^F_4_)7*s*_1/2_	99.8	0.2	7*s* ^4^F	95
	96614.352	7/2	96630	(^3^F_4_)7*s*_1/2_	99.9	0.1	7*s* ^2^F	66
	101199.085	5/2	101199	(^3^P_2_)7*s*_1/2_	97.6	2.4	7*s* ^2^D	43
N	101517.59	3/2	101500	(^3^P_2_)7*s*_1/2_	99.5	0.5	7*s* ^2^D	46
N	104090.70	7/2	104210	(^3^F_4_)6*d*_3/2_	0.9	99.1	6*d*(^3^F)^4^D	62
N	104410.05	11/2	104405	(^3^F_4_)6*d*_3/2_	0	100.	6*d*(^3^F)^2^H	46
J	104636.905	9/2	104612	(^3^F_4_)6*d*_3/2_	0.1	99.9	6*d*(^3^F)^4^F	36
	104763.45	13/2	104698	(^3^F_4_)6*d*_5/2_	0	100.	6*d*(^3^F)^4^H	95
N	104930.26	3/2	105955	(^3^F_4_)6*d*_5/2_	0.7	99.3	6*d*(^3^F)^2^P	58
	105066.347	11/2	105029	(^3^F_4_)6*d*_5/2_	0	100.	6*d*(^3^F)^4^G	72
N	105086.83	7/2	105046	(^3^F_4_)6*d*_5/2_	0.1	99.9	6*d*(^3^F)^2^F	61
	105388.130	9/2	105413	(^3^F_4_)6*d*_5/2_	0	100.	6*d*(^3^F)^2^G	48
N	105794.53	7/2	105739	(^3^F_3_)7*s*_1/2_	98.9	1.1	7*s* ^4^F	69
N	105962.52	5/2	105880	(^3^F_3_)7*s*_1/2_	99.0	1.0	7*s* ^4^F	59
J	106434.92	5/2	106430	(^3^F_4_)6*d*_5/2_	1.1	98.9	6*d*(^3^F)^2^D	39
N	109346.33	3/2	109412	(^3^P_2_)6*d*_3/2_	3.5	96.5	*6d*(^1^D)^2^D	37
N	109507.99	1/2	109472	(^3^P_2_)6*d*_3/2_	43.8	56.2	7*s* ^4^F	32
N	109527.87	5/2	109446	(^3^P_2_)6*d*_3/2_	3.3	96.7	6*d*(^1^D)^2^F	37
N	109676.18	7/2	109676	(^3^P_2_)6*d*_3/2_	0.2	99.8	6*d*(^1^D)^2^G	24
N	110020.85	1/2	110077	(^3^P_2_)6*d*_5/2_	10.7	89.3	6*d*(^1^D)^2^F	40
N	110146.80	7/2	110061	(^3^P_2_)6*d*_5/2_	0	100.	6*d*(^1^D)^2^F	23
N	110158.16	5/2	110261	(^3^F_2_)7*s*_1/2_	94.6	5.6	7*s* ^4^P	44
N	110257.49	9/2	110313	(^3^P_2_)6*d*_5/2_	0.1	99.9	6*d*(^1^D)^2^G	45
N	110258.18	3/2	110356	(^3^F_2_)7*s*_1/2_	93.9	6.1	7*s* ^4^F	42
N	110408.02	3/2	110530	(^3^F_2_)6*d*_5/2_	2.1	97.9	*6d*(^1^D)^2^P	39
N	111162.69	5/2	111075	(^3^P_2_)6*d*_5/2_	1.6	98.4	6*d*(^1^D)^2^D	32
N	111371.71	1/2	111309	(^3^P_0_)7*s*_1/2_	45.6	54.4	7*s* ^4^P	33
N	112433.31	3/2	112371	(^3^P_1_)7*s*_1/2_	90.9	9.1	7*s* ^4^P	65
N	113119.61	1/2	113112	(^3^P_1_)7*s*_1/2_	93.1	6.9	7*s* ^2^P	76
N	114127.60	9/2	114088	(^3^F_3_)6*d*_3/2_	0.1	99.9	6*d*(^3^F)^4^H	58
N	114256.30	7/2	114179	(^3^F_3_)6*d*_3/2_	0	100.	6*d*(^3^F)^4^G	48
N	114455.05	5/2	114530	(^3^F_3_)6*d*_3/2_	0.3	99.7	6*d*(^3^F)^4^D	40
N	114539.25	11/2	114549	(^3^F_3_)6*d*_5/2_	0	100.	6*d*(^3^F)^4^H	63
N	114861.32	9/2	114823	(^3^F_3_)6*d*_5/2_	0.1	99.9	6*d*(^3^F)^4^G	54
N	115060.84	5/2	115144	(^3^F_3_)6*d*_5/2_	0.2	99.8	6*d*(^3^F)^2^D	31
N	116689.04	9/2		5*d*^7^6*s*7*s*				
N	117340.84	9/2	117404	(^1^G_4_)7*s*_1/2_	98.7	1.3	7*s* ^2^G	94
N	117493.46	7/2	117437	(^1^G_4_)7*s*_1/2_	96.6	3.4	7*s* ^2^G	92
N	119057.05	5/2	a					
N	121651.19	9/2		5*d*^7^6*s*7*s*				

a Undetermined identification; theoretical *J* = 5/2 levels are calculated at 119011 and 119177 cm^−1^.

Notes: N—new energy level.

J—revised *J*-value.

**Table 5:** Odd energy levels of Pt II

	*E* (cm^−1^)	*J*	Configuration	Designation
	51408.370	7/2	5*d*^8^6*p*	(^3^F_4_)6*p*_1/2_
	53875.493	9/2	5*d*^8^6*p*	(^3^F_4_)6*p*_1/2_
	56587.934	3/2	5*d*^8^6*p*	(^3^P_2_)6*p*_1/2_
	57018.130	5/2	5*d*^8^6*p*	(^3^P_2_)6*p*_1/2_
	60907.688	9/2	5*d*^8^6*p*	(^3^F_4_)6*p*_3/2_
	61058.490	11/2	5*d*^8^6*p*	(^3^F4)6*p*_3/2_
	61190.026	5/2	5*d*^8^6*p*	(^3^F4)6*p*_3/2_^a^
	61665.485	7/2	5*d*^8^6*p*	(^3^F4)6*p*_3/2_
	62781.658	1/2	5*d*^8^6*p*	(^3^P_2_)6*p*_3/2_
	62820.489	9/2	5*d*^7^6*s*6*p*	(^4^F_9/2_,^3^P_0_)
	63738.841	7/2	5*d*^8^6*p*	(^3^F_3_)6*p*_1/2_
	64388.642	3/2	5*d*^8^6*p*	(^3^F_2_)6*p*_1/2_
	64757.343	5/2	5*d*^8^6*p*	(^3^F_3_)6*p*_1/2_^a^
N	65046.23	11/2	*5d*^7^6*s*6*p*	(^4^F_9/2_,^3^P_1_)
	65351.069	5/2	5*d*^8^6*p*	(^3^P_2_)6*p*_3/2_
	65587.115	1/2	5*d*^8^6*p*	(^3^P0)6*p*_1/2_
	66028.014	3/2	5*d*^8^6*p*	(^3^P_2_)6*p*_3/2_
	66434.315	7/2	5*d*^8^6*p*	(^3^P_2_)6*p*_3/2_
N	67780.44	7/2	*5d*^7^6*s*6*p*	(^4^F_9/2_,^3^P_1_)
J	69235.665	3/2	5*d*^8^6*p*	(^3^P_1_)6*p*_1/2_
	69953.317	5/2	5*d*^8^6*p*	(^3^F_3_)6*p*_3/2_
	70181.281	9/2	*5d*^7^6*s*6*p*	(^4^F_9/2_,^3^P_1_)
	70379.023	5/2	5*d*^8^6*p*	(^3^P_1_)6*p*_3/2_
N	71021.13	9/2	5*d*^8^6*p*	(^3^F_3_)6*p*_3/2_
J	71314.594	7/2	5*d*^8^6*p*	(^3^F_3_)6*p*_3/2_
N	71364.68	3/2	5*d*^8^6*p*	(^3^F_3_)6*p*_3/2_
	71948.916	5/2	5*d*^7^6*s*6*p*	
	73026.380	3/2	5*d*^8^6*p*	(^3^F_2_)6*p*_3/2_
	73431.346	9/2	5*d*^8^6*p*	(^1^G_4_)6*p*_1/2_
	73761.739	7/2		
E, J	73999.85	5/2		
	74241.479	3/2	5*d*^8^6*p*	
N	74409.47	11/2	5*d*^7^6*s*6*p*	(^4^F_9/2_,^3^P_2_)
	74619.107	5/2		
	74745.916	7/2		
	74754.823	½	5*d*^8^6*p*	(^3^F_2_)6*p*_3/2_
	75184.880	7/2	5*d*^8^6*p*	(^1^G_4_)6*p*_1/2_
N	75365.84	5/2		
	75581.422	3/2		
	76461.526	5/2		
	76610.046	3/2		
	77519.724	9/2		
N	77538.25	3/2		
N	77763.58	½		
E, J	78043.02	7/2		
N	78254.80	5/2		
N	78452.50	3/2		
	78906.492	9/2	*5d*^7^6*s*6*p*	(^4^F_9/2_,^3^P_2_)
	79092.09	3/2		
N	79607.460	5/2		
N	79683.41	1/2		
N	80197.33	7/2	*5d*^8^6*p*	(^1^G_4_)6*p*_3/2_
	80858.488	5/2		
N	81083.95	9/2	*5d*^8^6*p*	(^1^G_4_)6*p*_3/2_
E	81897.71	7/2		
E, J	82535.79	5/2		
N	82692.28	9/2	*5d*^7^6*s*6*p*	
E	82824.00	3/2		
E, J	82972.72	3/2		
	83352.251	7/2		
N	83538.53	11/2	5*d*^7^6*s*6*p*	
	84182.633	9/2		
E,J	85700.27	9/2	5*d*^7^6*s*6*p*	
N	85775.64	11/2		
N	85826.57	7/2		
N	86489.76	5/2		
N	87204.35	3/2		
N	88110.30	3/2,(5/2)		
N	88173.46	7/2		
N	88589.53	7/2		
N	89095.05	3/2		
	89607.936	5/2		
E	89863.27	9/2		
N	90173.25	7/2,9/2		
N	90746.64	5/2		
N	91016.64	1/2,3/2		
N	91271.16	5/2		
N	91669.95	7/2		
N	92526.90	11/2	5*d*^7^6*s*6*p*	
N	92537.08	9/2		
N	92749.02	3/2		
N	92767.97	3/2		
N	93197.46	5/2		
N	93322.18	11/2	5*d*^7^6*s*6*p*	
	93336.287	7/2		
	93482.013	7/2		
E	94022.39	9/2		
N	94271.53	5/2		
N	94633.25	5/2		
N	94829.73	1/2,3/2		
N	94842.49	5/2		
N	95226.00	9/2		
N	95557.71	3/2		
N	95617.03	7/2		
E	95754.07	5/2		
N	96109.73	11/2		
N	96131.24	9/2		
N	96403.32	5/2		
N	96443.92	1/2		
N	97183.40	3/2		
	97630.600	7/2		
N	97786.55	3/2		
N	97792.75	5/2		
J	98186.971	7/2		
	98817.744	7/2		
N	99068.74	3/2		
	99209.011	5/2		
N	99471.02	1/2		
	99797.778	5/2		
N	100232.63	9/2		
	100239.421	5/2		
	100611.695	3/2		
	100795.666	3/2		
	100903.454	7/2		
N	101113.06	1/2		
	101341.867	7/2		
N	101394.01	1/2		
	101397.850	5/2		
J	101549.10	9/2		
	101618.459	11/2		
	101916.930	5/2		
	102086.034	9/2		
	102414.857	5/2		
N	102520.80	7/2		
N	102613.05	11/2		
N	102678.30	3/2		
N	102872.20	7/2		
N	103060.55	9/2		
N	103421.16	3/2		
	103463.310	5/2		
	103517.132	7/2		
N	103637.26	1/2,3/2		
N	104092.10	3/2,5/2		
N	104158.64	5/2		
N	104548.13	3/2		
N	104625.27	9/2		
N	104831.58	9/2		
N	105018.17	3/2		
N	105042.36	5/2		
N	105554.33	7/2		
N	105597.33	5/2		
N	105726.12	1/2		
N	105896.50	3/2		
N	106229.90	7/2		
N	106852.84	3/2		
N	106995.20	9/2		
N	106996.55	7/2		
N	107191.45	3/2,5/2		
N	107386.26	9/2		
N	107588.13	3/2		
N	108037.26	7/2		
N	108038.05	3/2		
N	108155.51	3/2		
N	108322.40	7/2		
N	108639.24	5/2		
N	108672.51	1/2,3/2		
N	108727.50	7/2		
N	108802.20	7/2,9/2		
N	109307.89	7/2		
N	109528.23	3/2,5/2		
N	109733.10	7/2		
N	109753.67	5/2		
N	110066.71	9/2		
N	110085.70	3/2		
N	110196.40	5/2		
N	110202.39	7/2		
N	110609.08	9/2		
N	110638.00	5/2		
N	110684.45	3/2		
N	110762.77	7/2,9/2		
N	111320.57	5/2		
N	111354.67	7/2		
N	111716.41	7/2,9/2		
N	112247.69	9/2		
N	113785.71	3/2,(1/2)		
N	114880.48	5/2		

a These *J*-*j* characters are equally shared by the levels 61190 and 64757.

Notes: N—new energy level.

J—revised *J*-value.

E—revised energy value.
